# A New Decellularization Protocol of Porcine Aortic Valves Using Tergitol to Characterize the Scaffold with the Biocompatibility Profile Using Human Bone Marrow Mesenchymal Stem Cells

**DOI:** 10.3390/polym14061226

**Published:** 2022-03-17

**Authors:** Marika Faggioli, Arianna Moro, Salman Butt, Martina Todesco, Deborah Sandrin, Giulia Borile, Andrea Bagno, Assunta Fabozzo, Filippo Romanato, Massimo Marchesan, Saima Imran, Gino Gerosa

**Affiliations:** 1 Department of Cardiac, Thoracic, Vascular Sciences and Public Health, University of Padua, I-35128 Padua, Italy; marika.faggioli96@gmail.com (M.F.); salman.butt@studenti.unipd.it (S.B.); assunta.fabozzo@aopd.veneto.it (A.F.); gino.gerosa@unipd.it (G.G.); 2 L.I.F.E.L.A.B. Program, Consorzio per la Ricerca Sanitaria (CORIS), Veneto Region, I-35127 Padua, Italy; moroarianna97@gmail.com (A.M.); martina.todesco@unipd.it (M.T.); sandrin.deborah@gmail.com (D.S.); giulia.borile@unipd.it (G.B.); andrea.bagno@unipd.it (A.B.); filippo.romanato@unipd.it (F.R.); 3 Department of Industrial Engineering, University of Padua, I-35131 Padua, Italy; 4 Department of Physics and Astronomy “G. Galilei”, University of Padua, I-35131 Padua, Italy; 5 Consultant of Animal and Food Welfare, 3500 Padova, Italy; massimo.marchesan@yahoo.it

**Keywords:** decellularization, Tergitol, valve bioprostheses, cardiac tissue engineering, mesenchymal stem cells, biocompatibility

## Abstract

The most common aortic valve diseases in adults are stenosis due to calcification and regurgitation. In pediatric patients, aortic pathologies are less common. When a native valve is surgically replaced by a prosthetic one, it is necessary to consider that the latter has a limited durability. In particular, current bioprosthetic valves have to be replaced after approximately 10 years; mechanical prostheses are more durable but require the administration of permanent anticoagulant therapy. With regard to pediatric patients, both mechanical and biological prosthetic valves have to be replaced due to their inability to follow patients’ growth. An alternative surgical substitute can be represented by the acellular porcine aortic valve that exhibits less immunogenic risk and a longer lifespan. In the present study, an efficient protocol for the removal of cells by using detergents, enzyme inhibitors, and hyper- and hypotonic shocks is reported. A new detergent (Tergitol) was applied to replace TX-100 with the aim to reduce toxicity and maximize ECM preservation. The structural integrity and efficient removal of cells and nuclear components were assessed by means of histology, immunofluorescence, and protein quantification; biomechanical properties were also checked by tensile tests. After decellularization, the acellular scaffold was sterilized with a standard protocol and repopulated with bone marrow mesenchymal stem cells to analyze its biocompatibility profile.

## 1. Introduction

Cardiovascular diseases are the most common cause of mortality in high-income countries [1] and are estimated to be responsible for one in three deaths worldwide [2]. Amongst them, aortic stenosis (AS) is the most common heart valve lesion encountered in clinical practice and affects 2% to 5% of adults over the age of 65 years [3]. Furthermore, narrowing at various level of the left ventricle may also affect the pediatric population, which shows a prevalence of 0.22 per 1000 live births [4]. The currently available surgical intervention is the replacement of the diseased valve either with biological prostheses or mechanical devices. The major limitations associated with the former are short post-surgical lifespan (10 years) and low aptness in young patients [5,6]; the latter have longer durability but require lifelong anticoagulation therapy, which results in chronic complications.

Xenogeneic substitutes, i.e., chemically treated acellular valves, can be an alternative. Two main advantages are associated with these valves: first, their large availability; second, after implantation, they can stimulate mechanisms of tissue regeneration and healing, which favor specific cell types to grow and differentiate [7,8]. This is due to the presence of tissue-specific biochemical factors that are still preserved, even after decellularization. Indeed, the immunogenic responses triggered by biological tissues are still a hurdle that can be limited by improving decellularization protocols.

Decellularization involves the use of chemical/biological agents, physical stimulation, and/or a combination of both. Generally, decellularization protocols are applied through perfusion or under mechanical agitation to allow chemicals (mainly detergents, e.g., SDS, Triton, and sodium deoxycholate) or enzymes (e.g., trypsin) penetrating the tissue or the whole organ. Numerous studies demonstrated that decellularization is efficient for removing the DNA content, with an acceptable level of damage to the ECM composition, architecture, bioactivity, and mechanical properties [9,10]. The removal of nuclear materials is of primary importance since high levels of residual DNA can trigger severe immunological responses. Therefore, it is crucial to minimize the DNA content while avoiding potential structural damage to tissue/organ during decellularization [11].

In the present study, we propose a new efficient, low cytotoxic decellularization protocol (based on current data), which is based on a new detergent, Tergitol, which is eco friendly and highly degradable. The previously frequently applied Triton-100, is included in the candidate list by the European Chemical Agency (ECHA) because of its endocrine toxicity [12]; the alternative detergents were proposed by the same authorities, and we selected Tergitol due to its surfactant properties and rapid degradability. To our best of knowledge, the systemic toxicity of Tergitol has not been reported previously; however, it was reported to be toxic in the *C. elegans* model elsewhere [13]. The new decellularization protocol includes a cocktail of protease inhibitors, freezing and thawing cycles, and the application of Tergitol and sodium cholate in various steps along the whole process. Decellularization was applied to porcine aortic valves: the acellular matrices were characterized regarding their histological, immunological, biochemical, and biomechanical properties and shown to have significant perseverance.

ECMs are prone to cell attachment: this indicates scaffold biocompatibility and the possibility for tissue integration and remodeling in vivo. A well-preserved structure can provide a favorable environment for cell repopulation: the crosstalk between ECMs and cells can also promote the structural organization, directing cell functions. Understanding the process underlying the cross talk between cells and ECMs will lead to the design of the remodeling process of the engineered heart valves [14].

## 2. Materials and Methods

### 2.1. Tissue Procurement and Preservation

Fresh porcine hearts (20 in total) were harvested from 6–8 month-old adult pigs from the local abattoir (F.lli Guerriero S.r.l, Villafranca Padovana, Italy) within 2 h from death. The aortic roots were dissected from the heart and, after removing fat and blood remnants, washed with phosphate buffered saline (PBS, Sigma, Saint Louis, MO, USA), dried on blotting paper, and frozen in organ plastic bags at −80 °C until use.

### 2.2. Decellularization Procedure

The Tergitol-based decellularization protocol was evolved from the TRICOL protocol previously reported by our group [15]. Briefly, tissues were thawed at RT for 3 h, before initiating the decellularization procedure. Then, they were submerged in 500 mL sterile jars in agitation at 4 °C with proteases inhibitors cocktail for 8 h, afterwards with 1% Tergitol (Sigma) at RT for the next 12 h; hypertonic/hypotonic shocks (8 h each cycle at RT) followed, and finally they were treated with 0.4% sodium cholate (Sigma) for 16 h in the dark at RT. After decellularization, 2 cycles of washing with PBS (1X) were performed. Ethanol (4%, Carlo Erba, Cornaredo, Milano, Italy) and peracetic acid (0.1%, Sigma) solution was used for bioburden reduction. Finally, an endonuclease (Benzonase 25k U, Sigma, E1014) was applied to remove nucleic acids for 48 h at 37 °C.

### 2.3. DNA Extraction and Quantification

Native (*n* = 4) and decellularized tissues (*n* = 4) were lyophilized with the Speed vac SPD130DLX (Thermo Scientific). Dry tissue samples (5–10 mg each) were used for DNA extraction that was performed according to the procedures described in the DNeasy Blood & Tissue Kit (Qiagen^®^, 69506, Valencia, CA, USA). Briefly, tissues were incubated in proteinase K solution and the lysis buffer ATL was added overnight in thermomixer at 56 °C. DNA concentrations were measured by using Nanodrop One (Thermo Fisher Scientific, Waltham, MA, USA) and Qubit (Qubit^TM^ 1X dsDNA HS Assay Kit, Q33231, Waltham, MA, USA) fluorescent-based assays.

### 2.4. Biochemical Assays

Biochemical assays were performed to determine the concentration of major structural proteins (collagen and elastin) and glycosaminoglycans.

#### 2.4.1. Elastin Quantification

Native (*n* = 3) and decellularized (*n* = 3) aortic valves were dissected separately to investigate the myocardium, the aortic wall, and the cusps. Lyophilized samples (3–5 mg) were used for elastin quantification. Elastin concentration (mg/mg of dry weight of tissue) was measured according to the manufacturer’s instructions (Elastin Assay-Fastin, Biocolor, F4000): the assay is reported in [16]. Absorbance (513 nm) was measured with the Spark spectrophotometer ((Spark 10M, Tecan, Manne Dorf, Switzerland).

#### 2.4.2. Hydroxyproline Quantification

Samples (3–5 mg) of lyophilized native (*n* = 3) and decellularized (*n* = 3) valves were used for hydroxyproline quantification. The assay was carried out according to the manufacturer’s instructions (MAK008, Sigma-Aldrich, Saint Louis, MO, USA). The protocol was based on the principle established in [17], and the end-point absorbance (560 nm) was measured spectrophotometrically.

#### 2.4.3. Glycosaminoglycans (GAGs) Quantification

Lyophilized samples (3–5 mg) from myocardium, aortic wall and cusps (*n* = 6) were used for glycosaminoglycans quantification, according to the manufacturer’s instructions (Blyscan Sulfated Glycosaminoglycan Assay, Biocolor, B1000, County Antrim, UK). This assay is based on the protocol established in [18]; the end point absorbance (656 nm) was measured spectrophotometrically.

### 2.5. Biomechanical Tests

The effects on the aortic arterial wall and leaflets due to the decellularization procedure were biomechanically assessed by uniaxial tensile tests: native and decellularized aortic valve responses to load were compared. A total of 3 native and 3 decellularized valves were analyzed.

To evaluate the anisotropy of the aortic wall, 3 specimens were cut from the aortic wall in the longitudinal direction and 3 specimens in the circumferential direction [19]. The leaflets were isolated and cut into dog-bone-shaped specimens, using a homemade cutter. The dimensions and shape of each specimen respect what is suggested by the ASTM D1708-13 standard concerning small-size tissues: the gauge length was 5 mm, and the width 2 mm. Sample thickness was measured using Mitutoyo digital caliber model ID-C112XB (Aurora, IL, USA).

Samples were biomechanically assessed by uniaxial tensile loading tests performed with a custom-made apparatus (IRS, Padova, Italy), which was operated by a dedicated LabVIEW software (National Instruments, Austin, TX, USA).

The axial force was measured by means of a 50 N load cell, and the displacement between two actuators was taken as a direct measurement of sample elongation. Tests were performed at room temperature; samples were preloaded up to 0.1 N, then elongated with a rate of 0.2 mm/s until rupture. Displacement and load were recorded with a sampling frequency of 1000 Hz.

Biomechanical data were analyzed by means of an in-house developed Matlab^®^ script. For each sample, the stress–strain relationship was obtained, and the following parameters were calculated: engineering stress σ (MPa) as the tensile force measured by the loading cells (Newton) divided by the original cross-sectional area of the sample; strain ε (%) as the ratio between the grip displacement and the gauge length.

Since the stress–strain curve of soft tissues has a typical J-shape, it can be split into two regions with different stiffness. The first part of the curve is usually termed “elastin phase”, while the second is named “collagen phase”, to indicate the contribution of these proteins to the mechanical response to load. Tensile modulus E1 was calculated as the slope of the linear portion of the curve between 0% and 10% deformation. The modulus E2 was calculated between 60% and 100% deformation, to characterize the second portion of the curve.

The ultimate tensile strength (UTS) and failure strain (FS) values were also calculated, representing the maximum strength and maximum elongation of the sample, respectively. As a final parameter, toughness (I) was calculated: it represents the energy required for the sample to reach failure.

Statistical comparisons were performed using the *t*-test (GraphPad Prism Software, San Diego, CA, USA). Significance was set at *p* < 0.05.

### 2.6. Histology

Native (*n* = 6) and decellularized tissues (*n* = 6) were cut into pieces (5–8 mm) and embedded with OCT. Myocardium was fixed with 4% paraformaldehyde (PFA, Bioptica) followed by sucrose gradient (10% to 30%) before embedding with OCT under nitrogen vapors. All samples were frozen at −80 °C until analysis. Cryosections of each tissue (7–8 μm thick) were obtained using a cryostat (NX 70 HOMVPD Cryostar, Thermo Fisher Scientific, Waltham, MA, USA). Staining with hematoxylin and eosin (04-061010, BioOptica, Milan, Italy), Masson’s trichrome (04-01082, Bio-Optica, Milan, Italy), Weigert Van Gieson (04-051802, Bio-Optica, Milan, Italy) were performed according to each kits’ instructions. Images were acquired using EVOS XL Core Cell Imaging System (Thermo Fisher Scientific, Waltham, MA, USA) and processed with Fiji software (version 1.51n).

### 2.7. Immunofluorescence

Immunofluorescence was performed with antibodies (Table S3) to detect structural proteins in ECM. Primary antibodies included Collagen I, Collagen IV, Elastin, and Laminin; DAPI was applied for nuclear staining. Briefly, cryosections (7–8 μm thick) were fixed with 4% PFA, blocked with 1% of bovine serum albumin (BSA, Sigma) for 30 min at RT. Incubation with the primary antibodies at 4 °C in the dark in a humidified chamber was followed by secondary antibodies (Table S3) for 90 min at RT. After 3 washings with PBS (5 min each), samples were incubated with Nublu DAPI (Life Technologies, Thermo Fisher Scientific, Waltham, MA, USA) for 30 min at RT. After mounting the cryosections, images were taken with Leica CTR 6000. Post-imaging analysis was performed using Fiji software.

### 2.8. Determination of Alpha-Gal Epitope

Immunofluorescence was performed as described in the section material and method (immunofluorescence). Alpha-gal images were obtained with confocal microscope Axio Observer LSM 800 and processed with Fiji software.

### 2.9. Two Photon Microscopy

Native and decellularized samples (aortic wall, leaflets, and myocardium) were compared, acquiring the SHG signal to evaluate collagen I intensity and distribution and staining the tissues with DAPI (Life Technologies, Thermo Fisher Scientific, Waltham, MA, USA) to evaluate the presence of nuclei. Two-photon microscopy was performed by using a custom developed multiphoton microscope, previously described by Filippi et al. [20]. Briefly, an incident wavelength of 800 nm (~40 mW average laser power, under the microscope objective) was adopted to detect the collagen SHG signal at 400 nm, and DAPI was detected in the blue channel. Images were acquired at a fixed magnification through the Olympus 25X water immersion objective with 1.05 numerical aperture (1024 × 1024 pixels), averaged over 70 consecutive frames, with a pixel dwell time of 0.14 μs and a pixel width of 0.8 μm. First order analysis of collagen intensity and distribution was performed with dedicated FIJI plugins as reviewed in (https://www.mdpi.com/1422-0067/22/5/2657, accessed on 15 November 2021).

### 2.10. Scanning Electron Microscopy (SEM)

Patches of the aortic wall from native and decellularized valves were analyzed with SEM. Before the analysis, patches were stored in the fridge until the preparation for microscopy with an ascending scale of ethanol concentration.

Samples were analyzed by SEM in High Vacuum after fixing, dehydrating (with Ethanol), drying (with CPD Critical Point Dryer) and coating with Au.

### 2.11. Sterility Assessment

Sterilization of decellularized tissue was performed following the guidelines of the European Pharmacopoeia [21]. Decontamination of the decellularized samples was performed in two phases: first, samples were treated with 70% ethanol for 30 min at RT; afterwards they were treated with a cocktail of antibiotics and antimycotics (50 mg/L vancomycin hydrochloride (SBR00001, Merck, Kenilworth, NJ, USA), 8 mg/L gentamicin (G1397, Merck), 240 mg/L cefoxitin (C0688000, Merck) and 25 mg/L amphotericin B (A9528, Merck)) at 37 °C for 24 h.

For the sterility assessment, two specific liquid media (thioglycolate medium, Cat no. T9032, and soya-bean casein digest medium, Cat no. 22092, Sigma-Aldrich) were used, and turbidity was observed over time. The 8 mm punches of tissue samples (aortic wall, leaflets, and myocardium, each in duplicate) from native, decellularized, and eventually decontaminated valves were immersed into media to detect aerobic/anaerobic bacteria and fungal growth. Samples were incubated at 35 °C in the oven (Thioglycolate medium) and RT (Soya-bean casein digest medium) for 14 days. Images were taken during incubation at time intervals of 48 h.

### 2.12. Biocompatibility Test

Human bone marrow mesenchymal stem cells (hMSC-BM, 12974, PromoCell, Heidelberg, Germany) were cultured in bone marrow MSC media (C-28019, PromoCell). Cells were expanded and passaged up to 5 passages in T75 flasks in 5% CO_2_ at 37 °C with 95% of humidity. Decellularized and sterilized tissue patches (8 mm) were placed in 48-well plate and equilibrated with the MSC media for 24 h prior to culturing cells. Cells were harvested and resuspended in media with a density of 1 × 10^6^ cells/mL. Approximately 30,000 cells were plated over each tissue sample. The proliferation/toxicity analysis at each time point (24 h, 72 h, day 7, and day 14) was performed by the following protocols:

(a) Live and Dead assay: Cell viability was evaluated using the Live/Dead viability/cytotoxicity kit (MP 03224, Invitrogen, Thermo Fisher Scientific, Waltham, MA, USA). Briefly, cells were stained with Calcein AM and Ethidium homodimer-1 with final concentrations of 2 and 4 µM, respectively, by incubation at 37 °C for 45 min. Images were obtained using Olympus IX71 microscope.
(b) DNA extraction and quantification: Tissue patches were cultured with the hMSCs-BM and the amount of DNA was quantified to estimate cell proliferation. DNA extraction and quantification were performed as described in Section 2.3.
(c) WST-1 assay: The cell Proliferation and Cytotoxicity Assay kit (AR1159, Boster, Pleasanton, CA, USA) was used to assess the viability/proliferation of cells cultured on decellularized patches at each time point. The assay was performed according to the suppliers’ instructions. Absorbance was measured at 450 nm using microplate reader (Spark 10M Tecan, Tecan). Cells plated on a multi-well plate served as a positive control.
(d) Scanning electron microscopy (SEM): SEM was performed after culturing hMSCs-BM for 14 days on the decellularized and decontaminated aortic tissue patches (*n* = 3).

### 2.13. Data Analysis

All data were expressed as mean ± SD. One way ANOVA was performed with GraphPad Prism 8 to analyze the groups of experiments and multiple comparisons within groups were performed if necessary. Differences were considered statistically significant when *p* < 0.05.

## 3. Results

### 3.1. Visual Inspection

**Figure 1:**
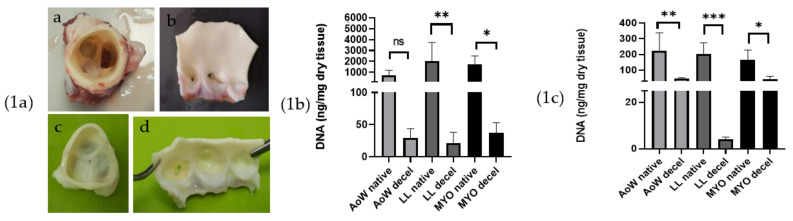
Macroscopic analysis of native (**a**,**b**) and decellularized (**c**,**d**) porcine aortic valve (**1a**). DNA quantification (ng/mg of dry tissue) comparative analysis on native and decellularized tissues performed by Nanodrop (**1b**) and Qubit assay (**1c**). Data were analyzed by one-way ANOVA (*p* < 0.05). There was a significant difference between native and corresponding decellularized tissues denoted by *, **, *** the level of significance from lower to highest *p* values, ns = not significant.

After decellularization, porcine valves appeared white, except for the myocardium, exhibiting a light brown color (Figure 1a).

The tissues were subgrouped according to the analysis to be performed. Tissues were lyophilized for the DNA quantification and biochemical assays. For biomechanical tests, tissue was taken fresh after decellularization with the defrosted native aortic valves. For the rest of the analysis, tissues were frozen in liquid nitrogen after embedding into the optimal cutting temperature (OCT). The results obtained are presented in the following section.

### 3.2. DNA Extraction and Quantification

After decellularization, the DNA quantities resulted in being below the threshold value of 50 ng/mg dry tissue [22]. Samples from the aortic wall and myocardium showed a DNA content between 40 and 50 ng/mg of dry tissue, while it was found to be between 3 and 10 ng/mg of dry tissue in leaflets (Figure 1b,c).

### 3.3. Biochemical Assays

**Figure 2:**
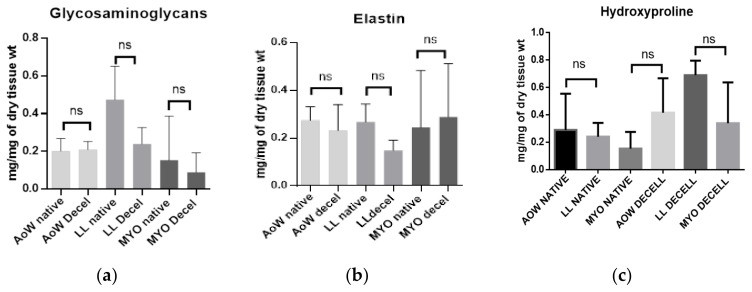
Biochemical analysis of (**a**) glycosaminoglycans, (**b**) elastin and (**c**) hydroxyproline shown. Native (*n* = 3) and decellularized (*n* = 3) samples were used for the analysis. Data were analyzed by one-way ANOVA (*p* < 0.05). There was a non-significant (ns) difference between native and corresponding decellularized tissues. Increasing values of hydroxyproline are not statistically significant.

Biochemical assays showed a non-significant loss of GAGs (Figure 2a) and elastin (Figure 2b) after decellularization. On the other hand, the hydroxyproline concentration increased, particularly in leaflets, but the increase was not significant (Figure 2c).

### 3.4. Biomechanical Tests

**Figure 3:**
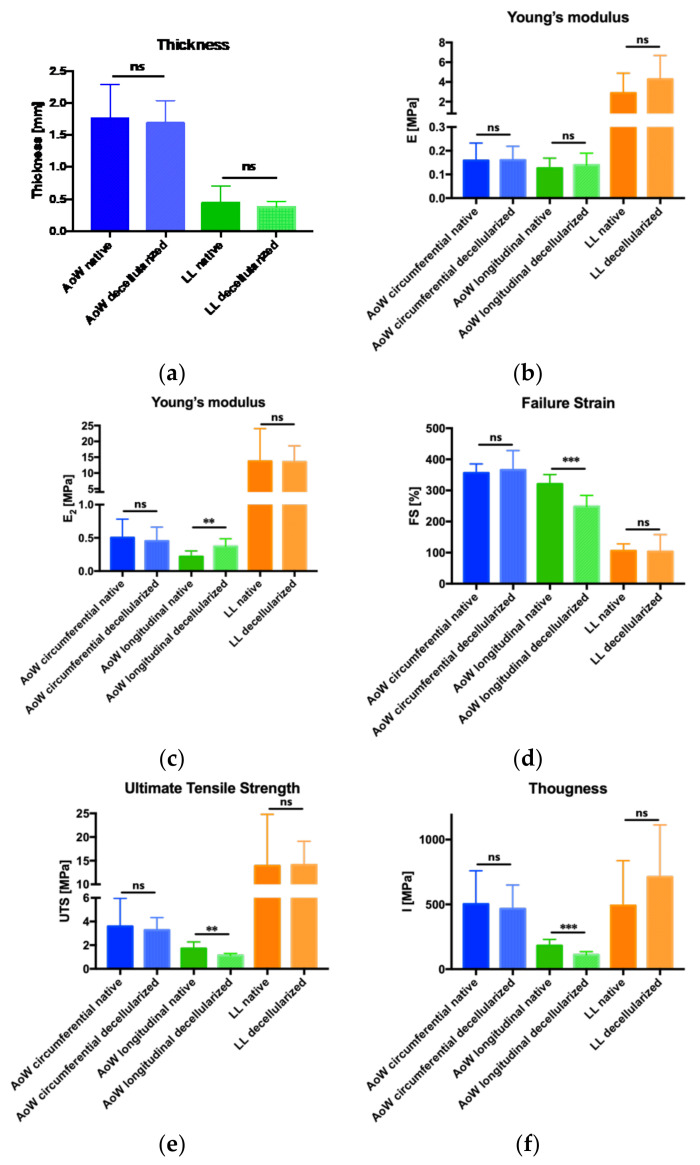
Histograms of mechanical tests. Sample thickness are not statistically different comparing native and decellularized samples (**a**). Modulus E1 values are not statistically different (**b**), whereas modulus E2 values are statistically different for the longitudinal aortic wall comparing samples before and after decellularization (**c**). These samples are statistically different also in terms of failure strain (**d**), ultimate tensile strength (**e**) and toughness (**f**) (ns: no significant different, ** *p* < 0.001, *** *p* < 0.001).

The decellularization treatment caused a decrease in the thickness of the aortic wall and leaflet: from 1.8 ± 0.52 mm to 1.7 ± 0.35 mm (5.6%), and from 0.45 ± 0.25 to 0.38 ± 0.09 (15.6%), respectively. Differences were not significant (Figure 3a, Table 1).

Concerning the parameters that characterize the mechanical behavior of the investigated tissues upon loading (Figure 3), there was an increase in stiffness along the longitudinal direction, which is significant in the E2 module (*p* = 0.0055) that characterizes the collagen part of the stress–strain curve. Other significant differences along the longitudinal direction are found with respect to FS (*p* = 0.0003), UTS (*p* = 0.0074) and I value (*p* = 0.0001): decellularized tissues reached lower elongation and tension at break than native ones. Indeed, FS values range from 320.5% ± 30.42% for native tissues to 248.2% ± 35.71% for decellularized ones, while UTS values vary from 1.71 ± 0.55 to 1.12 ± 0.18, respectively; the parameter I decreases in decellularized tissues from 181.9 ± 47.47 MPa to 110.6 ± 24.62 MPa.

### 3.5. Histology

**Figure 4:**
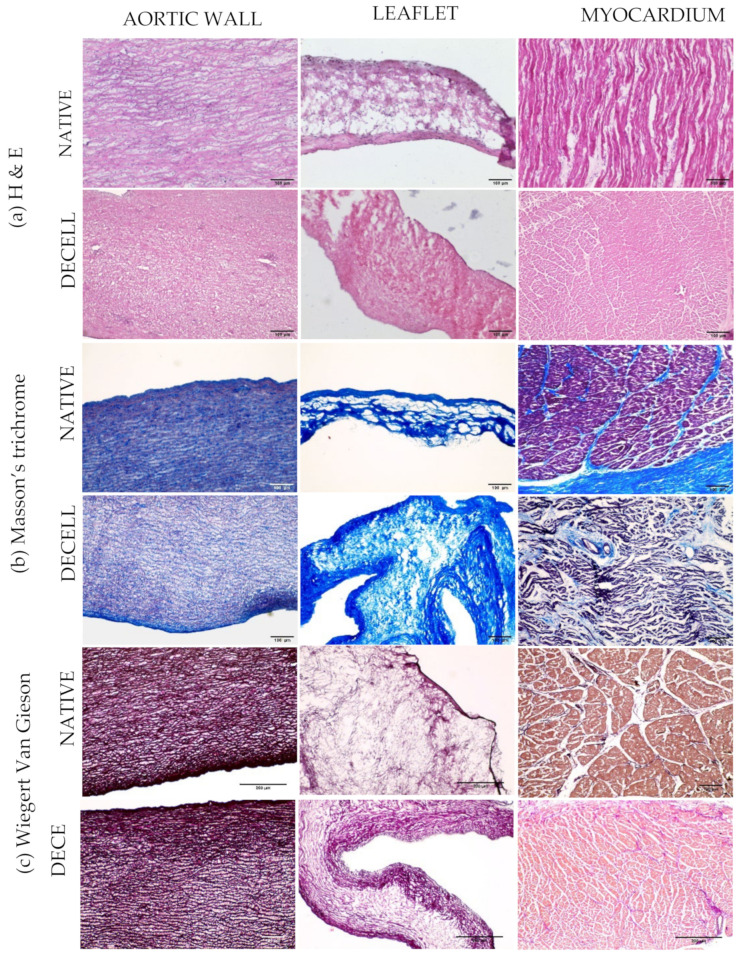
Histological analysis of native and decellularized tissues was performed on OCT embedded cryosections (7 µm). (**a**) Hematoxylin and Eosin staining showed the presence of nuclei in native aortic wall, leaflet, and myocardium and the removal of nuclei in the decellularized counterparts. (**b**) Masson’s trichrome showed the nuclei in black staining aligned with collagen in blue. (**c**) Wiegert Van Gieson staining indicates the presence of nuclei in native tissues (elastin and collagen are in red) while nuclei are absent in the decellularized tissues.

In general, histological analyses showed the integrity of the ECM, even after decellularization. In the representative pictures (Figure 4), the collagen and elastin arrangement were found to be intact, with a well-preserved fiber distribution that was confirmed by TEM; nuclei were effectively removed, and they were not more visible in the decellularized samples.

### 3.6. Immunofluorescence

**Figure 5:**
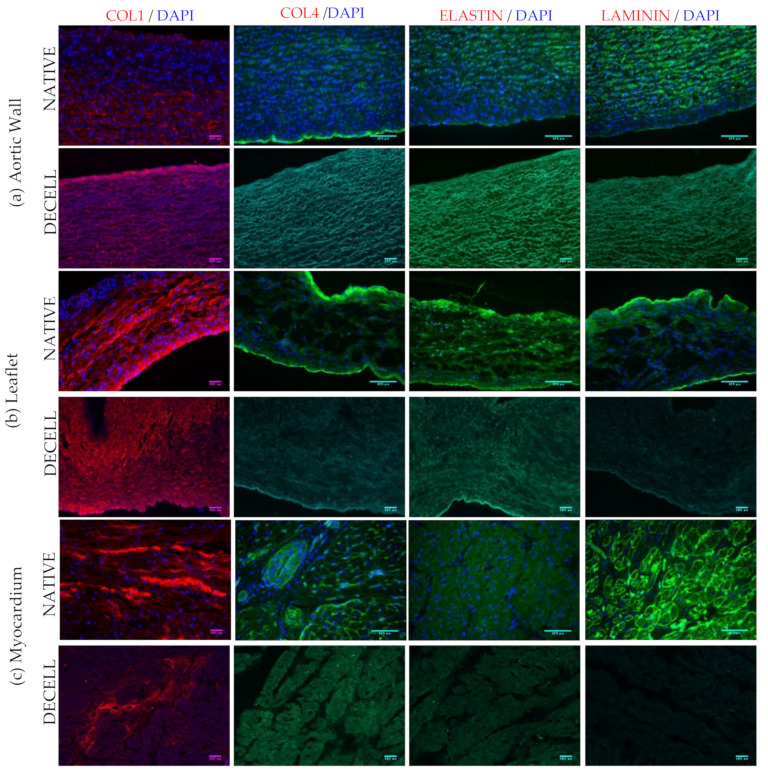
Immunofluorescence analysis showed the PFA fixed cryosections stained with primary and secondary antibodies and DAPI (blue). (**a**) aortic wall, (**b**) leaflets, and (**c**) myocardium. The representative images indicate the expression of collagen I (red), collagen IV (green), elastin (green) and laminin (green). Images were taken with the Leica imaging system at 20× magnification.

Immunofluorescence was used to evaluate ECM structure and decellularization effectiveness. DAPI staining showed the presence of nuclei in native samples while their absence was observed in decellularized tissues. Regarding the expression of collagen I, collagen IV, laminin and elastin, no significant difference was detected between native and decellularized aortic walls; in decellularized leaflets and myocardium, reduced signals of laminin and collagen IV were found (Figure 5).

With regards to the alpha-gal epitope, which is major immunogenic factor in animal tissues, signals were significantly maintained comparing native and decellularized samples. However, detection with isolectin showed a reduction in the decellularized myocardium (Figure S1).

### 3.7. Two Photon Microscopy

To deepen the investigation of structural proteins, with a peculiar interest in collagen, we evaluated Collagen I in a semi-quantitative approach thanks to SHG imaging. Compared to immunofluorescence staining that requires a processing of the tissues, SHG offers a label-free approach to acquire the collagen I signal, proportional to the protein content. Moreover, samples were stained with DAPI.

**Figure 6:**
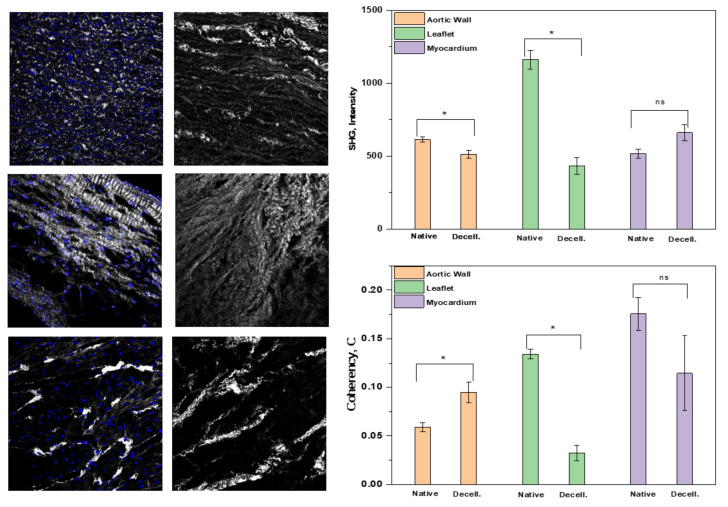
Pictures obtained by means of two photon microscope from native and decellularized aortic wall, leaflets and myocardium (ns = non significant, * denoted significance of difference between values of native and decellularized values).

In Figure 6, representative images of native and decellularized tissues from the aortic wall, leaflet, and myocardium are shown. The DAPI signal is completely absent in decellularized portions. This observation further confirms the effective nuclei removal, thanks to the decellularization protocols. The first-order analysis of the SHG signal is focused on the average intensity that is proportional to the protein content. We observed a decrease in signal intensity in the aortic wall and leaflets after decellularization, while no changes appeared in the myocardium samples. In line with this, a re-arrangement of the collagen fibers resulted from coherency analysis for the aortic wall and leaflets. Again, no differences were observed in myocardium when comparing native and decellularized tissue.

### 3.8. Scanning Electron Microscopy (SEM)

**Figure 7:**
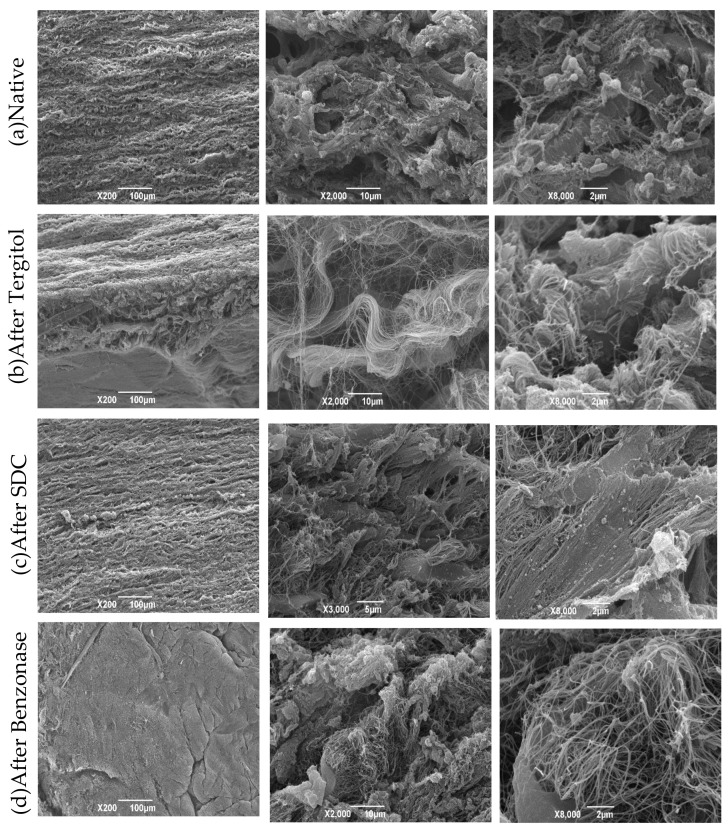
SEM images represent the gradual changes in morphological features of the native aortic wall tissue (**a**) and along the sequential steps of decellularization (**b**–**d**).

Samples from the aortic root were analyzed at different steps all along the decellularization process: native, after Tergitol, sodium cholate, Benzonase, and after 14 days from cell seeding with hMSCs-BM (Figure 7). Images of native tissue showed the presence of cells and the typical structure of collagen fibers inside the matrix. After Tergitol treatment, cells appeared in a lower amount or completely absent, while the collagen structure was slightly disrupted. After sodium cholate treatment, pictures showed the presence of cellular debris while collagen fibers were maintained: less tissue disruption is caused during this step. After Benzonase, nuclear components are completely absent.

### 3.9. Sterility Assessment

From the visual inspection of native tissue samples (Figure S2), turbidity clearly indicated contamination within 24–72 h decellularized samples were eventually turbid on day 7. Sterilized samples showed the absence of turbidity during an incubation time of 14 days.

### 3.10. Biocompatibility Assessment

**Figure 8:**
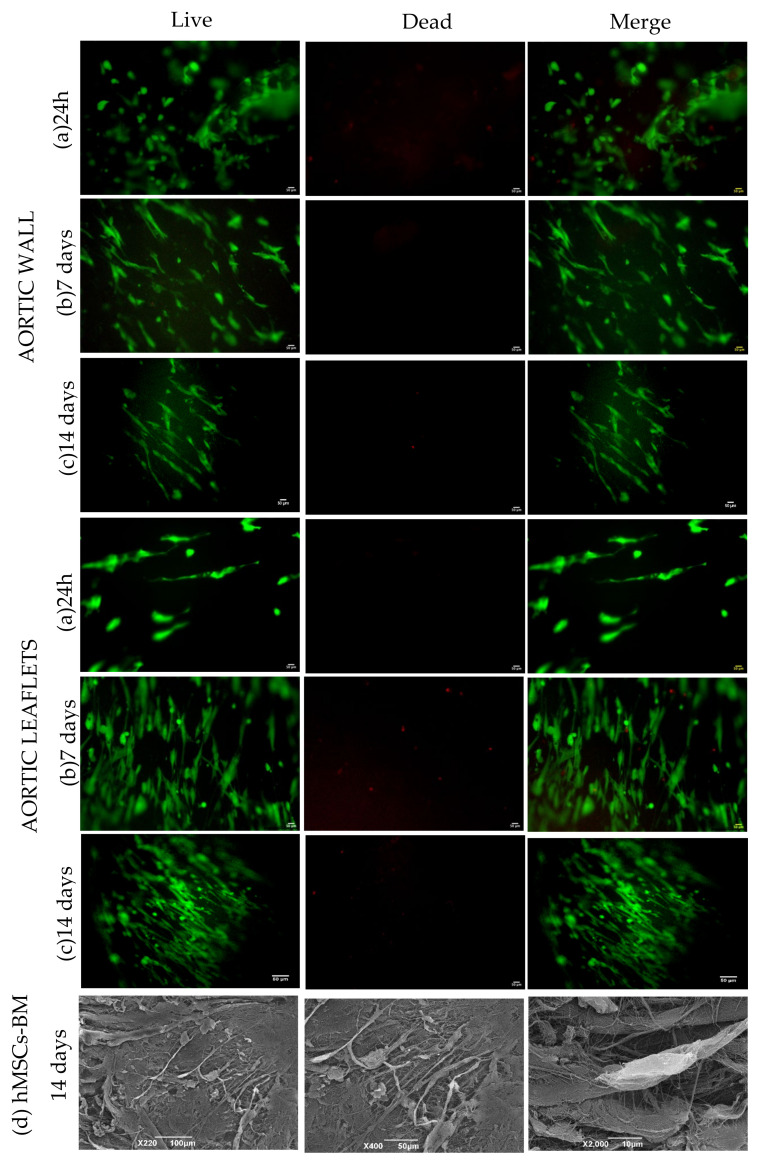
Live and dead staining analysis at (**a**) 24 h, (**b**) day 7 and (**c**) day 14 for aortic wall and leaflets samples. Images were taken with the Olympus IX7l microscope at 2× magnification. (**d**) SEM images of the decellularized tissue cultured with hMSCs-BM (day 14) represented with red arrows. Pictures are shown in different scale of magnification (see the scale bars).

Live and dead staining showed that the cells were attached to the tissue surface within 24 h: they seemed to be more prone to attach at the edges of the aortic patches, whereas they spread over the entire surface of the leaflets (Figure 8). Cell proliferation over both tissues was observed progressively until day 7: their elongation and alignment were oriented by tissue structure. On day 14, the cell viability on the aortic wall was compromised significantly, while the proliferation on the leaflet was sustained (Figure 8).

**Figure 9:**
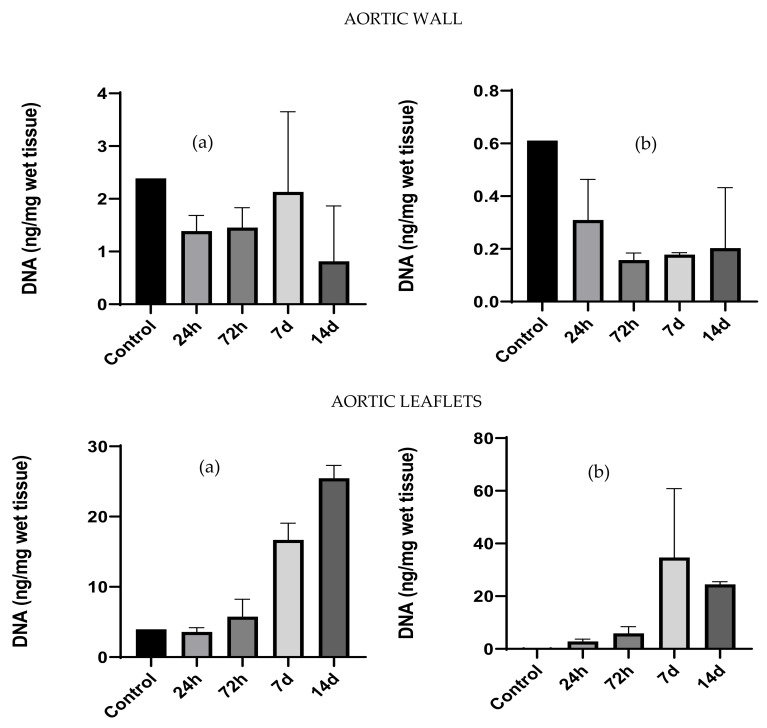
DNA quantification (ng/mg of dry tissue) comparative analysis between native and decellularized tissues performed by Nanodrop (**a**) and Qubit assay (**b**). Data were analyzed by one-way ANOVA (*p* < 0.05). There was a significant difference between native and corresponding decellularized tissues.

DNA content in cell cultured tissues progressively increased over time in the case of aortic leaflets, while in the aortic wall, there was a significant decrease on day 14 (Figure 9).

**Figure 10:**
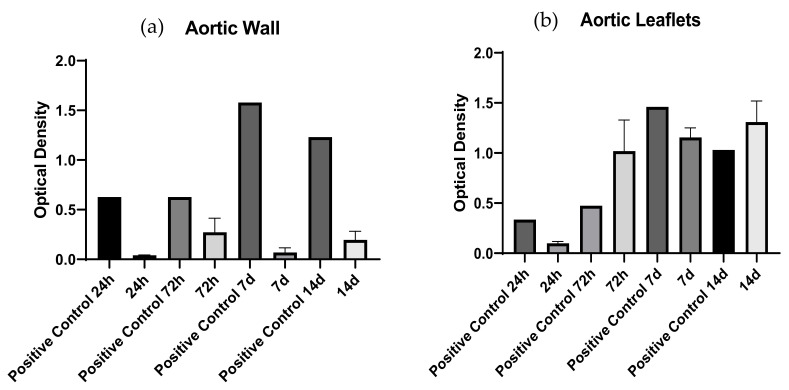
Analysis of metabolic activities of the cultured hMSC-BM cells at different time intervals was measured by WST-1 reduction assay. (**a**) aortic wall and (**b**) leaflets samples show significant differences in the proliferation capacity of the cells. Data were analyzed by one-way ANOVA (*p* < 0.05). There was a significant difference between native and corresponding decellularized samples for the aortic wall.

WST-1 assay is an indicator of the metabolic activity of living cells: the absorbance of the aortic wall samples was lower than the control (Figure 10). On the contrary, we observed a continuous absorbance increase over time in the leaflets (Figure 10), which was approximately equal to the positive control.

Scanning electron microscopy (SEM) was performed after culturing hMSCs-BM for 14 days, high magnification images depicted the cells attached to the surface of the aortic wall.

## 4. Discussion

Decellularized porcine heart valves can be exploited as substitutes for the surgical replacement of diseased valves: they present fundamental advantages over currently available mechanical and bioprosthetic devices since they do not evoke any adverse response and can be repopulated by circulating cells in vivo. The decellularization has to suppress the immunogenic potential of animal valves while preserving their structure and composition.

In the present paper, we illustrated a new decellularization protocol that is based on the previously reported one [15]. Major modifications include the use of a different detergent (Tergitol at room temperature instead of Triton X-100), freeze/thawing cycles, and reduced time points for certain inhibitor cocktail cycles. The Tergitol-based protocol was applied to porcine aortic valves (3 cm diameter), and it was demonstrated to be effective in removing cellular and nuclear components while preserving tissue structural integrity.

Histological analyses allowed excluding the presence of nuclei and confirmed substantial maintenance of collagen and elastin inside the ECM of decellularized samples. In particular, immunological assays revealed a minimal loss of collagen IV and laminin; microscopic observations showed that the nuclear material was efficiently removed with the persistence of collagen fibers.

With regard to the alpha-gal epitope, its removal seemed to be not effective: only in decellularized myocardium samples was it significantly less abundant than in native ones.

The determination of the DNA content in acellular biological scaffolds is of paramount importance to assess the effectiveness of decellularization protocol: previous studies have shown that residual DNA is still present in biological scaffolds already exploited for both research and commercial purposes [23,24]. In the present work, a maximal level of DNA was removed (≥95–97%) and the residual DNA amount was measured in valve leaflets (~10 ng/mg). We also compared two quantification methods: Nanodrop spectrophotometer assay exhibited a sensitivity that is lower than fluorescent-based Qubit assay.

Hydroxyproline is a diagnostic amino acid for collagen: thus, its concentration was calculated as an indicator of collagen amount. No significant difference was found comparing native and decellularized tissues. On the other hand, a loss of elastin was observed in all tissues after decellularization, but it was not significant: this evidence is a positive indicator for tissue suitability. The little increase in the hydroxyproline content of the tissue after decellularization was mainly due to a relative increase in the ratio of these molecules to the total dry weight, due to the loss of soluble proteins and cell components. A non-significant reduction in GAGs was also observed after decellularization. The ECM proteins quantification data revealed that the ECM integrity was found to be in a highly acceptable range. This presented the aortic tissue suitability for further study for the animal implantation research trials.

With regard to the biomechanical characterization performed by means of tensile tests, the decellularization process did not cause significant differences with regard to the stiffness values of tissue samples from both longitudinal and circumferential aortic walls and from leaflets, as they were measured in the first part of the stress–strain curves (modulus E1). Moreover, in the second part of these curves, where the contribution of collagen fibers withstanding the mechanical deformation becomes prominent, a significant increase in the E2 modulus of the longitudinal aortic wall samples appeared: thus, the decellularization process makes the tissue stiffer along the longitudinal direction. This result is confirmed by the other statistical differences evidenced in Figure 3: they only regard the comparison of the aortic wall samples along the longitudinal direction, before and after decellularization. As the E2 modulus increases, the failure strain and the ultimate tensile strength decreases: the first represents the ability of the tissue to be elongated upon load; the second is the maximin value of the load applied until failure. Actually, the decellularization process is responsible for a significant improvement in the stiffness along the longitudinal direction of the aortic wall samples, which is followed by a significant reduction in their extensibility and ability to resist the application of a mechanical load. Toughness (I) is the last biomechanical parameter shown in Figure 3: it is calculated as the area below the stress–strain curve, and its physical meaning is related to the amount of energy absorbed by the material upon deformation. Once again, the decellularization process caused a reduction in toughness only for the samples withdrawn for the longitudinal direction of the aortic walls.

In terms of the biocompatibility profile, it is worthwhile to mention that two different surfaces showed a difference in the growth of hMSCs-BM: the leaflets surface was found to be the most appropriate to allow cell growth. The ECMs are prone to cell attachment, indicating scaffold biocompatibility and the possibility for tissue integration and remodeling in vivo. A well-preserved structure can provide a favorable environment for cell repopulation. There could be a possibility that the cross talk between the ECMs and the cells could also be promoting the structural organization, directing the cell function. Understanding the process underlying the cross talk between the cells and the ECMS will lead to designing the remodeling process of the engineered heart valves.

## 5. Conclusions

At present, acellular xenogeneic valve conduits are applied as scaffolds for heart valve regeneration, with promising results [25,26,27]. Due to the toxicity related to the detergent used in the previous protocols, it is necessary to find appropriate alternatives. In the present study, Tergitol was applied to decellularize aortic conduits, which were then characterized following standardized criteria. From the results obtained, we suggest the exploitation of acellular heart valve conduits for studies on the animal model. Preclinical studies will be a step ahead toward the application of decellularized heart valves in humans as an alternative to the currently available prosthetic devices.

## Figures and Tables

**Table 1:** Sample thickness measure in Native and Decellularized. Aortic Wall (AoW) and Native Leaflet (LL).

Sample	Thickness [mm]
AoW Native	1.8 ± 0.52
AoW Decellularized	1.7 ± 0.35
LL Native	0.45 ± 0.25
LL Decellularized	0.38 ± 0.09

## Data Availability

Not applicable.

## Supplementary Materials

The following supporting information can be downloaded at: https://www.mdpi.com/article/10.3390/polym14061226/s1, Figure S1: Immunofluorescence data for the expression of α-gal; Figure S2: Sterility assessment; Tables S1 and S2: Biomechanical data; Table S3: List of antibodies.
