# Supplementary material for: A New Decellularization Protocol of Porcine Aortic Valves Using Tergitol to Characterize the Scaffold with the Biocompatibility Profile Using Human Bone Marrow Mesenchymal Stem Cells
Source: Polymers (Basel). 2022 Mar 17;14(6):1226. doi: 10.3390/polym14061226 (PMC8949722; doi:10.3390/polym14061226)
Supplement: Supplementary file 1 [file polymers-14-01226-s001.zip › polymers-1627369-supplementary.pdf]

polymers

MDPI

Supplementary Materials

A New Decellularization Protocol of Porcine Aortic Valves Using Tergitol to Characterize the Scaffold with the Biocompatibility Profile Using Human Bone Marrow Mesenchymal Stem Cells

Marika Faggioli [1,2], Arianna Moro [2,3], Salman Butt [1,2], Martina Todesco [2,3], Deborah Sandrin [2,4], Giulia Borile [2,4], Andrea Bagno [2,3], Assunta Fabozzo [1,2], Filippo Romanato [2,4], Massimo Marchesan [5], Saima Imran [1,2,*] and Gino Gerosa [1,2]

Citation: Faggioli, M.; Moro, A.; Butt, S.; Todesco, M.; Sandrin, D.; Borile, G.; Bagno, A.; Fabozzo, A.; Romanato, F.; Marchesan, M.; et al. A New Decellularization Protocol of Porcine Aortic Valves Using Tergitol to Characterize the Scaffold with the Biocompatibility Profile Using Human Bone Marrow Mesenchymal Stem Cells. *Polymers* **2022**, *14*, 1226. https://doi.org/10.3390/polym14061226

Academic Editors: Antonia Ressler and Inga Urlic

Received: 21 February 2022
Accepted: 14 March 2022
Published: 17 March 2022

Publisher's Note: MDPI stays neutral with regard to jurisdictional claims in published maps and institutional affiliations.

Copyright: © 2022 by the authors. Submitted for possible open access publication under the terms and conditions of the Creative Commons Attribution (CC BY) license (https://creativecommons.org/licenses/by/4.0/).

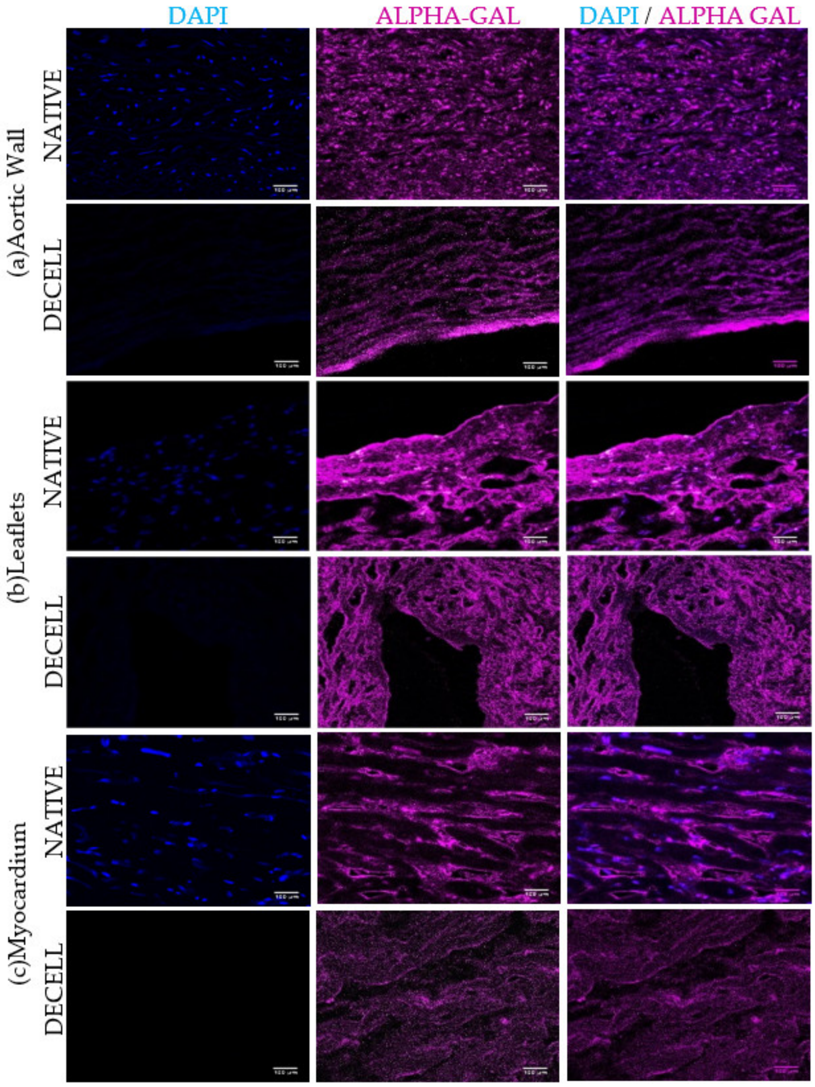

Figure S1. Immunofluorescence images for the expression of α-gal. Confocal imaging was performed using microscope Zeiss Axio Observer LSM 800 at 20× magnification. Comparative signals between native and decellularized tissues differ in intensity. The specificity of Alpha-gal is confirmed with the staining of Isolectin fluorophore. Images were processed with Fiji software.

Tables S1 and S2: Biomechanical data.

sample	E [MPa] (1-10%)	E_2 [MPa] (60-100%)	FS [%]	UTS [MPa]	I [MPa]
AoW circumferential native	0.15 ± 0.07	0.50 ± 0.28	356.1 ± 29.23	3.59 ± 2.37	502.4 ± 257.2
AoW circumferential decellularized	0.16 ± 0.05	0.45 ± 0.2	365.7 ± 62.33	3.26 ±1.067	465.2 ± 184.6
AoW longitudinal native	0.13 ± 0.04	0.21 ± 0.8	320.5 ± 30.42	1.71 ± 0.55	181.9 ± 47.47
AoW longitudinal decellularized	0.14 ± 0.05	0.37 ± 0.11	248.2 ± 35.71	1.12 ± 0.18	110.6 ± 24.62
LL native	2.88 ± 2.02	13.73 ± 10.35	105.6 ± 22.64	13.9 ± 10.9	490.9 ± 346.9
LL decellularized	4.27 ± 2.142	13.5 ± 5.16	103.2 ± 54.38	14.08 ± 5.01	711.6 ± 401.8

sample	E [MPa] (1-10%)	E_2 [MPa] (60-100%)	FS [%]	UTS [MPa]	I [MPa]
AoW circumferential native	0.15 ± 0.07	0.50 ± 0.28	356.1 ± 29.23	3.59 ± 2.37	502.4 ± 257.2
AoW circumferential decellularized	0.16 ± 0.05	0.45 ± 0.2	365.7 ± 62.33	3.26 ±1.067	465.2 ± 184.6
AoW longitudinal native	0.13 ± 0.04	0.21 ± 0.8	320.5 ± 30.42	1.71 ± 0.55	181.9 ± 47.47
AoW longitudinal decellularized	0.14 ± 0.05	0.37 ± 0.11	248.2 ± 35.71	1.12 ± 0.18	110.6 ± 24.62
LL native	2.88 ± 2.02	13.73 ± 10.35	105.6 ± 22.64	13.9 ± 10.9	490.9 ± 346.9
LL decellularized	4.27 ± 2.142	13.5 ± 5.16	103.2 ± 54.38	14.08 ± 5.01	711.6 ± 401.8

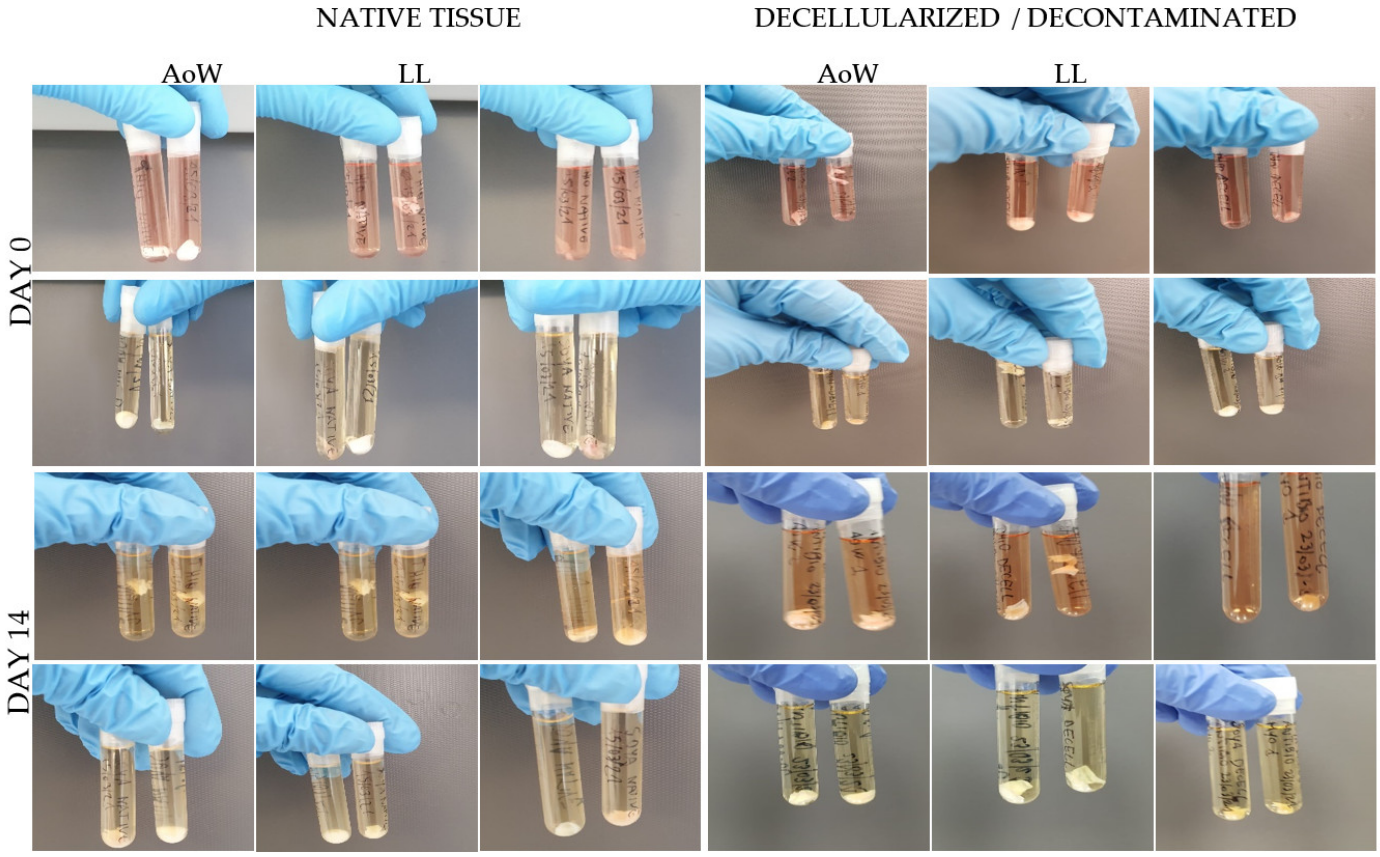

Figure S2. Sterility assessment of native and decellularized plus decontaminated (with Antibiotic and Antimycotic solution) samples from day 0 to day 14.

Table S3. List of the antibodies used in the study.

ANTIBODY	CAT. NUMBER	COMPANY	DILUTION
Anti-Collagen I	C2456	Sigma	Mouse, 1:100
Anti-Collagen IV	AMab6586	Abcam	Rabbit, 1:200
Anti-Elastin	AB2161D	Abcam	Rabbit, 1:50
Anti-Laminin	L9393	Sigma	Rabbit, 1:100
Anti-Alpha-gal	LS-C63415	LS Bio	1:10
DAPI	R37606	ThermoFischer Scientific	10μg/ml
Alexa Fluor 488 (anti-Rabbit)	A11008	Invitrogen by ThermoFisher	1:200
Alexa Fluor 555 (anti-Mouse)	A21422	Invitrogen by ThermoFischer	1:300
